# *Gas6* is a reciprocal regulator of mitophagy during mammalian oocyte maturation

**DOI:** 10.1038/s41598-019-46459-3

**Published:** 2019-07-17

**Authors:** Kyeoung-Hwa Kim, Eun-Young Kim, Jung-Jae Ko, Kyung-Ah Lee

**Affiliations:** Institute of Reproductive Medicine, Department of Biomedical Science, College of Life Science, CHA University, Pangyo-Ro 335, Bundang-gu, Seongnam-si, Gyeonggi-do 13488 Korea

**Keywords:** Mitophagy, Oogenesis, RNAi

## Abstract

Previously, we found that the silencing of growth arrest-specific gene 6 (*Gas6*) expression in oocytes impairs cytoplasmic maturation through mitochondrial overactivation with concurrent failure of pronuclear formation after fertilization. In this study, we report that *Gas6* regulates mitophagy and safeguards mitochondrial activity by regulating mitophagy-related genes essential to the complete competency of oocytes. Based on RNA-Seq and RT-PCR analysis, in *Gas6*-silenced MII oocytes, expressions of mitophagy-related genes were decreased in *Gas6*-silenced MII oocytes, while mitochondrial proteins and *Ptpn11*, the downstream target of *Gas6*, was increased. Interestingly, GAS6 depletion induced remarkable MTOR activation. *Gas6*-depleted MII oocytes exhibited mitochondrial accumulation and aggregation caused by mitophagy inhibition. *Gas6*-depleted MII oocytes had a markedly lower mtDNA copy number. Rapamycin treatment rescued mitophagy, blocked the increase in MTOR and phosphorylated-MTOR, and increased the mitophagy-related gene expression in *Gas6*-depleted MII oocytes. After treatment with Mdivi-1, a mitochondrial division/mitophagy inhibitor, all oocytes matured and these MII oocytes showed mitochondrial accumulation but reduced *Gas6* expression and failure of fertilization, showing phenomena very similar to the direct targeting of *Gas6* by RNAi. Taken together, we conclude that the *Gas6* signaling plays a crucial role in control of oocytes cytoplasmic maturation by modulating the dynamics and activity of oocyte mitochondria.

## Introduction

Growth arrest-specific gene 6 (GAS6) is a ligand of receptor tyrosine kinases of the TAM (Tyro3, Axl, and Mertk) receptors, and its role as a potential therapeutic target in human cancer has been recently emphasized^1^. The expression of *Gas6* is widespread in many tissues and cells, including immune cells, endothelial cells, vascular smooth muscle cells, bone marrow cells, adipocytes, platelets and various cancer cells^1–3^. GAS6 and TAM receptors activate a series of different downstream signaling cascades and regulate diverse functions, especially cell migration, adhesion, inflammation, cell growth, survival and other cell type-specific functions^4–6^.

To date, two studies support *Gas6* involvement in reproduction^7,8^. We first found that *Gas6* is expressed in mouse oocytes and is not involved in the completion of the meiotic division since *Gas6*-depleted oocytes mature to the metaphase II (MII) stage with extrusion of the first polar body^7^. However, depletion of *Gas6* in oocytes results in failure of sperm chromatin remodeling and pronuclear formation through insufficient cytoplasmic maturation^7^. In a successive study, we found that the depletion of *Gas6* in oocytes inhibited heparan sulfate biosynthesis and glutathione production via mitochondrial overactivation, characterized by increased ATP production and mitochondrial membrane potential^8^. Most importantly, *Gas6* was a crucial factor for the completion of oocyte cytoplasmic maturation.

Mitochondria are important cellular organelles for energy production. They have important roles in ATP synthesis, metabolism, calcium homeostasis, fatty acid oxidation and apoptosis^9^. Mitochondria play pivotal roles in mammalian reproduction. Researchers have reported that, in mice, mitochondrial activity is important for oocyte maturation and subsequent embryo development but that an altered mtDNA copy number does not affect nuclear maturation^9,10^. In humans, mitochondrial function and mtDNA contents are also correlated with oocyte maturity and fertilizability^11,12^. Moreover, fertilization failure observed in oocytes with a low mtDNA number and mitochondrial dysfunction was resulted from defective oocyte cytoplasmic maturation and decreased oocyte quality. Thus, the mtDNA copy number in MII oocytes is being investigated as a marker of embryo viability^13^. It has been reported that an increase in the number of healthy mitochondria and/or improvement in mitochondrial function could provide a significant boost in fertility^14,15^. Recently, mitochondrial replacement therapy has resulted in the birth of healthy primate (including human) offspring^16,17^.

As females become older, maternal reproductive capacity decreases. The main reason for this decline is the decreased quality of oocytes and their mitochondria. In oocytes, ATP production and mtDNA copy number decrease and mitochondrial mutations increase with aging^18^. Transgenic mice with induced mtDNA mutations exhibit reduced fertility^19^. In mammalian cells, mitochondrial quality can be improved through the facilitation of mitophagy and selective autophagy of mitochondria^20^. The clearance of dysfunctional mitochondria via mitophagy screens the damaged mtDNA and prevents its transmission to the next generation^21^.

Mitophagy is essential for the mitochondrial quality and quantity control mechanism that eliminates damaged mitochondria^22^. Removal of damaged mitochondria through mitophagy requires five steps: initiation of phagophore formation, elongation, closure and autophagosome formation, autophagosome-lysosome fusion and lysosomal degradation^23^. Autophagosome formation is finely regulated by autophagy-related genes^24^. Among the numerous proteins involved in the regulation of autophagy, MTOR is a key component. MTOR inhibits autophagy as well as mitophagy by decreasing the expression of autophagy-related genes^25^. Several studies have examined the role of the MTOR-specific inhibitor rapamycin in rescuing the poor developmental capacity of aged pig oocytes^26^ and cloned mouse and pig embryos^27,28^.

In this study, we compared gene expression profiles using RNA-Seq between oocytes with or without *Gas6* expression. Based on the data of the present study, we report for the first time that *Gas6* regulates mitophagy via a MTOR-dependent pathway during *in vitro* maturation of mouse oocytes. Additionally, *Gas6*-silenced MII oocytes exhibited the accumulation and aggregation of mitochondria in the cytoplasm. We found that *Gas6* depletion led to a greater suppression of mitophagy through MTOR signaling activation and a reduction in the mtDNA copy number and levels of mitochondria-encoded mRNA in oocytes, supporting a novel role of *Gas6* in the maintenance of mitochondrial contents and activity during oocyte cytoplasmic maturation.

## Results

### Definition of the transcriptome in *Gas6*-silenced MII oocytes

Previously, we found that *Gas6* depletion impairs cytoplasmic maturation and pronucleus (PN) formation^7^. We asked whether cytoplasmic maturity in oocytes is reflected at the level of transcription, and, if so, what patterns of gene expression are characteristic of *GFP*-dsRNA treated (control group) or *Gas6*-silenced MII oocytes. We performed transcriptome analysis with RNA-Seq. In this study, 18,666,312 to 41,440,784 raw reads were generated for each sample. Of these, 7,674 and 11,118 expressed genes were identified in *GFP*-dsRNA treated or *Gas6*-silenced MII oocytes, respectively. There were 2,238 commonly expressed genes between the two groups. A comparison of transcript abundances showed relatively few differentially abundant transcripts in MII oocytes between *GFP* RNAi and *Gas6* RNAi. A total of 312 genes were changed more than two-fold in *Gas6*-depleted MII oocytes than in *GFP* dsRNA-injected MII oocytes. This analysis revealed that 210 genes were upregulated and 102 were downregulated. The top 20 differentially expressed genes are listed in Table S1.

### Disruption of *Gas6* causes changes in mitochondria-related gene expression

**Figure 1:**
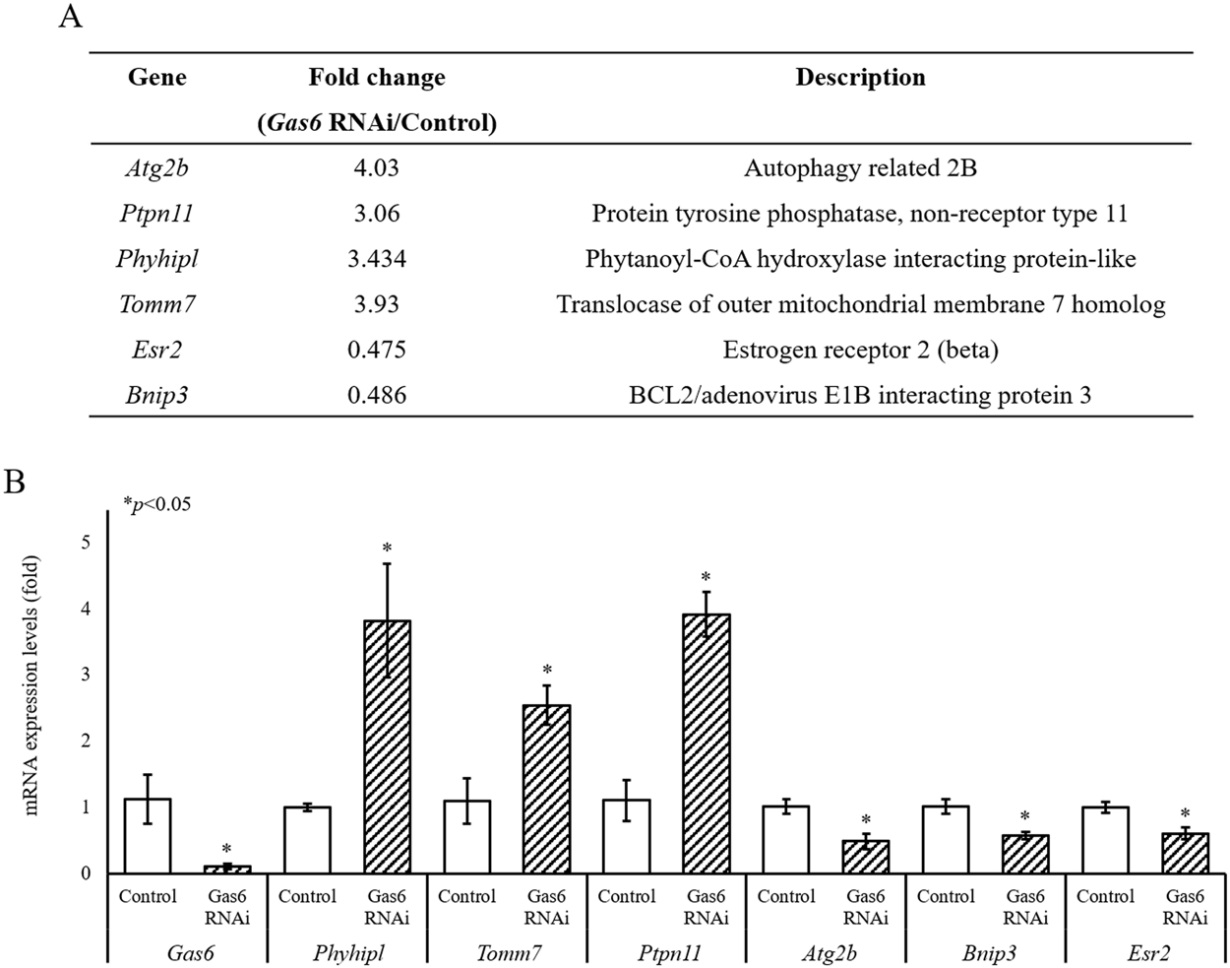
Validation of RNA-Seq and examination of the abundance of transcripts of mitochondria-related genes by qPCR. (**A**) Based on the RNA-Seq analysis, among the transcripts of differentially expressed genes, six genes that were involved in mitochondrial proteins (e.g., *Phyhipl* and *Tomm7*), downstream target of *Gas6* (*Ptpn11*) and mitophagy (e.g., *Atg2b*, *Binp3* and *Esr2*) were selected. (**B**) Changes in the expression of selected genes in *Gas6* dsRNA-injected MII oocytes were measured by qPCR. The experiments were performed in biological triplicates of pools of 20 oocytes, and the data are expressed as the mean ± SEM. Expression levels were calculated from C_T_ values after normalization with *H1foo*. Control, *GFP* dsRNA-injected MII oocyte; *Gas6* RNAi, *Gas6* dsRNA-injected MII oocyte. The asterisks represent statistical significance at *p* < 0.05.

Previously, we reported that disruption of *Gas6* resulted in insufficient oocyte cytoplasmic maturation via mitochondrial dysfunction such as mitochondrial overactivation and abnormal mitochondrial accumulation^8^. Based on the RNA-Seq analysis, we selected 2 genes related to mitochondrial functions (*Phyhipl* and *Tomm7*) and 3 for mitophagy (*Atg2b*, *Binp3* and *Esr2*) and added *Ptpn11*, as a downstream target of *Gas6* (Fig. 1A) for validation by quantitative real-time RT-PCR (qPCR). Genes associated with increased (*Atg2b*, *Ptpn11*, *Phyhipl* and *Tomm7*) or decreased (*Esr2* and *Bnip3*) transcript levels were evaluated by qPCR to determine the transcript levels in MII oocytes after *GFP* RNAi or *Gas6* RNAi. Five of the six genes analyzed demonstrated similarly significant changes in transcript levels between RNA-Seq and qPCR results, demonstrating the validity of RNA-Seq analysis; however, *Atg2b* showed an increase in transcript abundance in RNA-Seq but a decrease in expression by qPCR analysis (Fig. 1). Thus, transcript levels of mitochondrial proteins (*Phyhipl* and *Tomm7*) and *Ptpn11* were significantly increased, whereas transcript levels of mitophagy-related genes (*Atg2b*, *Binp3* and *Esr2*) were markedly reduced (Fig. 1B), suggesting that downregulation of the *Atg2b*, *Binp3* and *Esr2* genes may cause the accumulation of mitochondria as observed by us previously in *Gas6*-depleted oocytes.

### Disruption of *Gas6* induced the activation of MTOR signaling in oocytes

Based on RNA-Seq and qPCR results, we hypothesized that the mitochondrial dysfunction and inhibition of mitophagy through *Gas6* silencing may have resulted in failure of PN formation. It has been reported that MTORC1 plays a pivotal role in autophagy and can be selectively inhibited by rapamycin^29^. MTORC1 consists of MTOR, RAPTOR, mLST8 (GβL) and PRAS40^30^. Among these, MTOR is an important regulator of mitophagy, with activated MTOR suppressing mitophagy, and negative regulation of MTOR promoting mitophagy^25^. Therefore, we performed *Gas6* RNAi and then evaluated the changes in mitochondrial quality and quantity, expression of mitophagy-related genes, and MTOR activation in MII oocytes.

**Figure 2:**
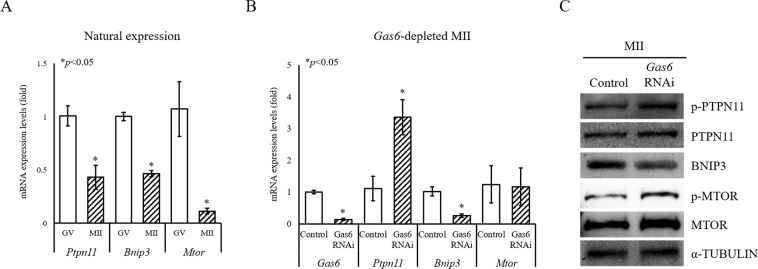
Depletion of *Gas6* induces activation of the PTPN11 and MTOR pathways. (**A**) Typical expression pattern of *Gas6* downstream target genes in GV and MII oocytes. For the PCR, a single oocyte-equivalent mRNA was used as a template for the amplification of each gene. GV and MII oocytes were harvested after *in vitro* culture for 0 and 16 hours, respectively. Expression levels were calculated from C_T_ values after normalization with *H1foo*. The asterisks represent statistical significance at *p* < 0.05. (**B**) The qPCR analysis of *Gas6* downstream target genes in *Gas6*-silenced MII oocytes, including *Ptpn11*, *Bnip3* and *Mtor*. Expression levels were calculated from C_T_ values after normalization with *H1foo*. Control, *GFP* dsRNA-injected MII oocyte; *Gas6* RNAi, *Gas6* dsRNA-injected MII oocyte. The asterisks represent statistical significance at *p* < 0.05. (**C**) Western blot analysis of p-PTPN11, PTPN11, BNIP3, p-MTOR and MTOR in *Gas6*-depleted MII oocytes. After *Gas6* RNAi, expression levels of p-PTPN11, PTPN11, p-MTOR and MTOR were increased, whereas the expression of BNIP3 was decreased. A protein lysate from 200 MII oocytes was loaded into each lane. α-TUBULIN was used as a loading control.

Previously, we found by using JC-1 staining that *Gas6* depletion induces mitochondrial overactivation and accumulation in the cytoplasm^8^. We hypothesized that the abnormal mitochondrial accumulation in response to decreased *Gas6* may be due to the activation of PTPN11 and MTOR as well as reduction of BNIP3, known as an inactivator of MTOR^31^. Thus, we next investigated the mechanism whereby *Gas6* regulates mitophagy, by measuring *Ptpn11*, *Bnip3* and *Mtor* in *Gas6*-silenced MII oocytes. The natural expression of *Gas6* downstream target genes, *Ptpn11*, *Bnip3* and *Mtor* was higher in GV than in MII oocytes (Fig. 2A). In *Gas6*-silenced MII oocytes, *Ptpn11* expression increased, whereas *Bnip3* expression was decreased (Fig. 2B). The expression of *Mtor* was not significantly altered (Fig. 2B). Interestingly, however, depletion of *Gas6* induced a remarkably increased expression of proteins, for PTPN11, phosphorylated-PTPN11 (p-PTPN11), MTOR and phosphorylated-MTOR (p-MTOR; Fig. 2C). These data suggest that, as we hypothesized, *Gas6* disruption leads MTOR activation probably through PTPN11 activation and BNIP3 suppression, which then may lead mitophagy inhibition.

### Suppression of mitophagy was observed after *Gas6* RNAi

**Figure 3:**
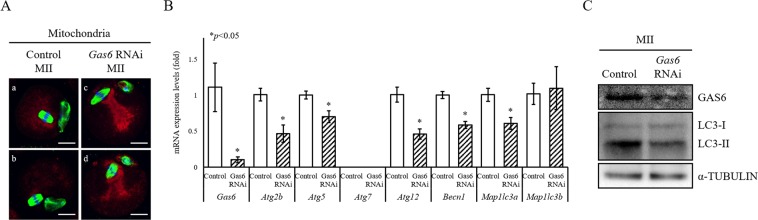
*Gas6* RNAi resulted in mitochondrial accumulation by mitophagy inhibition. (**A**) Abnormal mitochondrial distribution and accumulation were found in *Gas6*-silenced MII oocytes. After *GFP* (a and b) and *Gas6* (c and d) dsRNA was injected, MII oocytes were precultured in M16 medium containing MitoTracker (mitochondria, red) for 30 minutes and then stained with an antibody against α-TUBULIN (green). Oocytes’ DNA were also stained with DAPI (blue) and imaged by an LSCM. The scale bars indicate 20 μm. (**B**) The mRNA expression profiles of autophagy-related genes in MII oocytes were measured by qPCR analysis. Control, *GFP* dsRNA-injected MII oocyte; *Gas6* RNAi, *Gas6* dsRNA-injected MII oocyte. The asterisks represent statistical significance at *p* < 0.05. (**C**) Western blot analysis of LC3 conversion (LC3-I to LC3-II) in MII oocytes after *Gas6* RNAi treatment. Depletion of GAS6 protein after *Gas6* RNAi resulted in a reduction of LC3-II. A protein lysate from 200 MII oocytes was loaded into each lane. α-TUBULIN was used as a loading control.

As we illustrated in Fig. 2, *Gas6* RNAi inhibited the expression of genes related to mitophagy as well as the activation of MTOR signaling in oocytes. To investigate whether loss of *Gas6* promotes mitochondrial accumulation through mitophagy suppression and altered distribution in the cytoplasm; we stained mitochondria with MitoTracker. In the control *GFP* RNAi group, MII oocytes had evenly dispersed mitochondria in the cytoplasm, and some more-aggregated mitochondria around the spindles (Fig. 3Aa–b). Conversely, mitochondria were mainly accumulated and aggregated from the meiotic spindle to the cortex in the *Gas6*-silenced MII cytoplasm (Fig. 3Ac–d). To determine whether disruption of *Gas6* attenuates mitophagy in oocytes via the regulation of autophagy-related genes, we measured the changes in the expression of seven genes related to mitophagy, i.e., *Atg2b*, *Atg5*, *Atg7*, *Atg12*, *Becn1*, *Map1lc3a* and *Map1lc3b*. Among these genes, *Atg7* was not detected in oocytes (Fig. 3B). In *Gas6*-silenced MII oocytes, the expression levels of five autophagy-related genes, *Atg2b*, *Atg5*, *Atg12*, *Becn1* and *Map1lc3a*, were slightly decreased, while the expression of *Map1lc3b* was not changed (Fig. 3B). We next determined the expression of LC3 in *Gas6*-silenced MII oocytes. The LC3 are known as the mitophagy regulator marker. Indeed, the initiation of mitophagy causes the conversion of LC3-I to LC3-II, which is associated with the formation of autophagosomes. We measured LC3-II protein levels by Western blotting, and as shown in Fig. 3C, the LC3-II levels were markedly decreased after *Gas6* RNAi, suggesting decreased formation of autophagosomes. Taken together, these results suggest that *Gas6* depletion induced mitochondrial accumulation and altered the mitochondrial distribution in the cytoplasm probably via suppression of mitophagy.

### *Gas6* RNAi resulted in a lower mtDNA copy number

**Figure 4:**
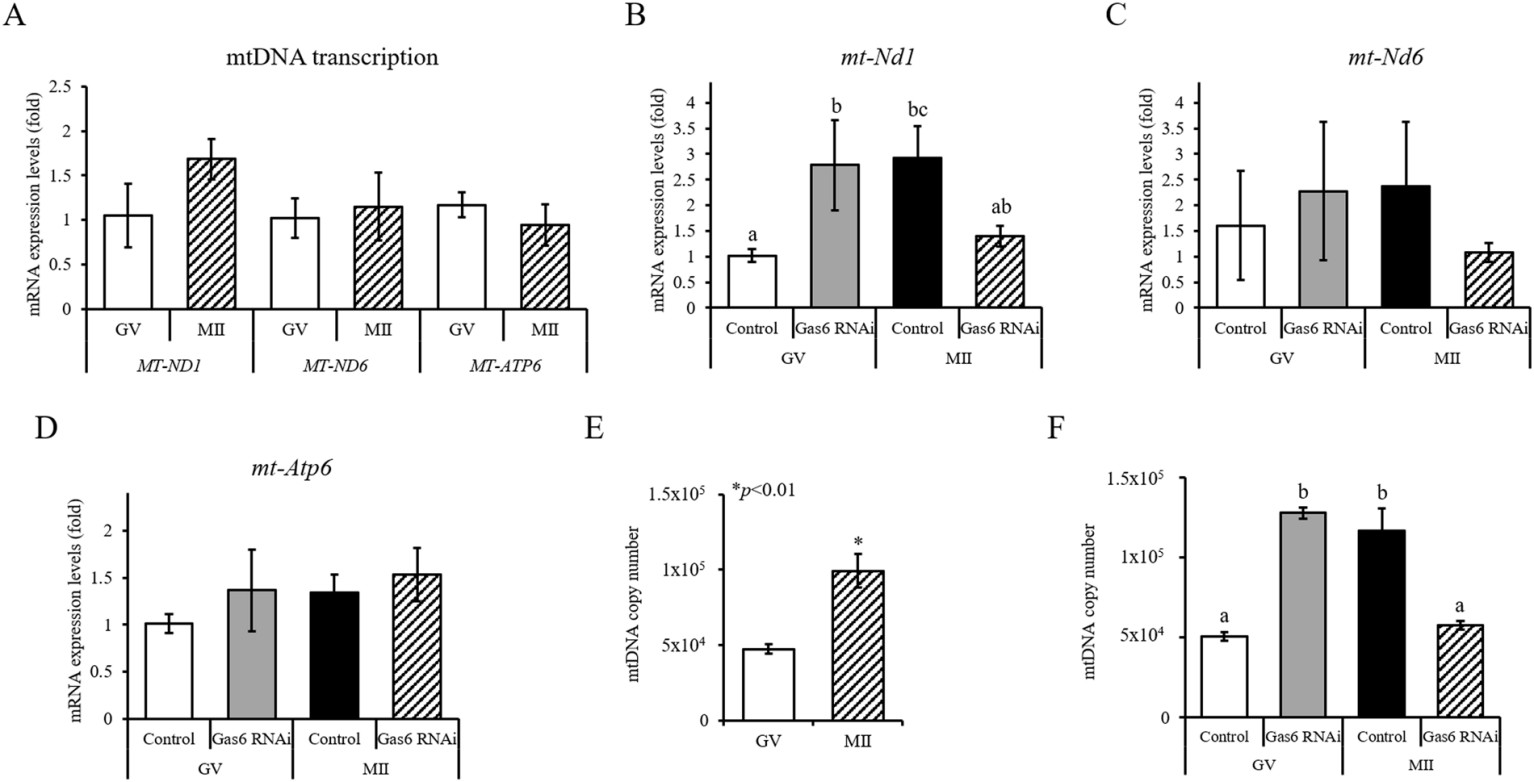
*Gas6* RNAi treatment caused a loss of mtDNA and impaired mitochondrial transcription. (**A**) Typical expression pattern of mtDNA-encoded genes during oocyte maturation. The data are presented as the mean ± SEM. (**B–D**) Expression of *mt-Nd1* (**B**), *mt-Nd6* (**C**), and *mt-Atp6* (**D**) in *Gas6*-silenced GV and MII oocytes. The transcript level of mtDNA-encoded genes was measured via qPCR after *Gas6* RNAi. Control, *GFP* dsRNA-injected MII oocyte; *Gas6* RNAi, *Gas6* dsRNA-injected MII oocyte. Different letters (a~c) indicate significant differences at *p* < 0.05. (**E**) Measurements of the mtDNA copy number during mouse oocyte maturation. The mtDNA copy number was higher in MII oocytes than in GV oocytes. The asterisk represents statistical significance at *p* < 0.01, as determined by a paired *t*-test. (**F**) *Gas6* disruption had opposite effects on the mtDNA content depending on the oocyte stage. GV, *Gas6* dsRNA-injected oocytes cultured in IBMX supplement for 8 hours; MII, *Gas6* dsRNA-injected oocytes cultured in IBMX supplement for 8 hours followed by 16 hours of culture in plain M16 medium. Different letters (a and b) indicate significant differences at *p* < 0.05.

Mitochondria play important roles in oocyte maturation^9^. We examined the expression of mRNAs associated with mitochondria during oocyte maturation. The expression of mtDNA-encoded genes, *mt-Nd1*, *mt-Nd6* and *mt-Atp6*, were not significantly altered during oocyte maturation (Fig. 4A). After *Gas6* RNAi, the expression of *mt-Nd1* was increased in GV but reduced in MII oocytes (Fig. 4B), but no change in *mt-Nd6* and *mt-Atp6* expression was observed (Fig. 4C and D).

Oocyte quality is tightly linked to the oocyte mtDNA content in mice, humans and other species^10–13^. To determine whether there was a change in the mtDNA copy number during *in vitro* maturation, qPCR was performed on GV and MII oocytes. We found that the GV oocytes contained an average of 47,400 ± 3,010 mtDNA copies per oocyte, while the MII oocytes possessed an average of 99,380 ± 11,070 mtDNA copies (Fig. 4E). The mtDNA copy number was almost doubled with oocyte maturation. In contrast, in Fig. 4F, the average mtDNA copy number was markedly lower in *Gas6*-depleted MII oocytes (57,590 ± 2,890) than in *Gas6*-silenced GV oocytes (127,950 ± 3,610), suggesting that *Gas6* disruption had opposite effects on the mtDNA content depending on the oocyte stage. Regarding the up-regulated mtDNA content in *Gas6*-silenced GV oocytes (Fig. 4F), we do not have substantial data for that specific phenomena at this moment. However, we guess the potential mechanism for that increase as follows. To allow *Gas6* dsRNA to work for breakdown endogenous *Gas6* transcripts at GV stage, we holding the GV membrane breakdown by treating oocytes with IBMX for 8 hours. It may be one of the potential points to check with. To hold the GV membrane intact against the natural breakdown force, oocytes may need more energy than normal maturation. That is the reason for increased mtDNA at GV stage after RNAi. However, more experiment is required for this issue.

### Inhibition of MTOR rescued impaired mitophagy after *Gas6* RNAi

**Figure 5:**
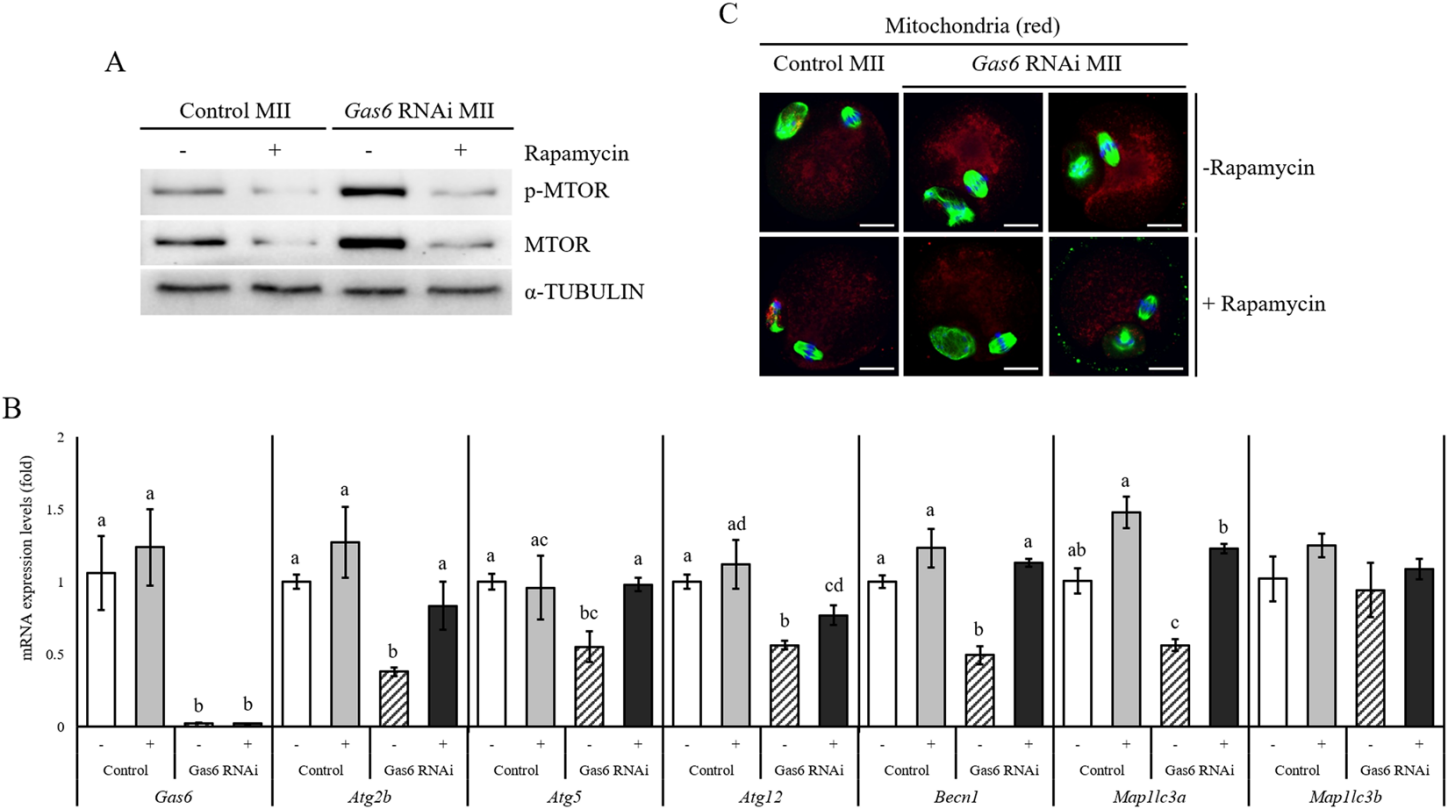
Blocking MTOR signaling rescued the inhibition of mitophagy caused by *Gas6* reduction. (**A**) The treatment with rapamycin reduced MTOR and p-MTOR protein levels in control and *Gas6*-silenced MII oocytes. A protein lysate from 150 MII oocytes was loaded into each lane. α-TUBULIN was used as a loading control. -, Non-treated; +, Rapamycin treated. (**B**) The mRNA expression profiles of autophagy-related genes were measured by qPCR analysis after *Gas6* RNAi with rapamycin treatment. Different letters (a~c) indicate significant differences at *p* < 0.05. (**C**) Inactivation of MTOR with rapamycin resulted in reduced mitochondrial content, disappearance of mitochondrial aggregates (red staining) in *Gas6* silencing oocytes. After rapamycin treatment, MII oocytes were precultured in M16 medium containing MitoTracker (mitochondria, red) for 30 minutes and then stained with an antibody against α-TUBULIN (green). Oocyte DNA was also stained with DAPI (blue), and the oocytes were imaged by an LSCM. The scale bars indicate 20 μm.

Having shown that the reduction of *Gas6* accumulates mitochondria in the cytoplasm via mitophagy suppression, we examined whether the MTOR-dependent regulatory pathway of mitophagy is related to this phenomenon in *Gas6*-silenced MII oocytes. To determine whether inhibition of the MTOR pathway would rescue impaired mitophagy by *Gas6* silencing, we added rapamycin, a MTOR inhibitor, to the medium for *in vitro* maturation after *Gas6* RNAi. Upon treatment, rapamycin-treated *Gas6*-silenced MII oocytes exhibited markedly reduced expression of MTOR and p-MTOR, indicating that rapamycin treatment blocked the MTOR activation caused by *Gas6* silencing (Fig. 5A). In addition, as shown in Fig. 5B, the expression levels of five autophagy-related genes, *Atg2b*, *Atg5*, *Atg12*, *Becn1* and *Map1lc3a*, were significantly upregulated in *Gas6*-depleted MII oocytes with rapamycin treatment. As expected, blocking MTOR signaling with rapamycin led a more even distribution of mitochondria throughout the cytoplasm with significantly reduced mitochondrial numbers (Fig. 5C; red staining). Importantly, rapamycin treatment rescued the mitophagy suppression caused by *Gas6* depletion, and so it was reasonable to conclude that the oocytes exhibited blocked MTOR activation and increased expression of autophagy-related genes.

### *Gas6* is a reciprocal regulator of oocyte mitophagy

**Figure 6:**
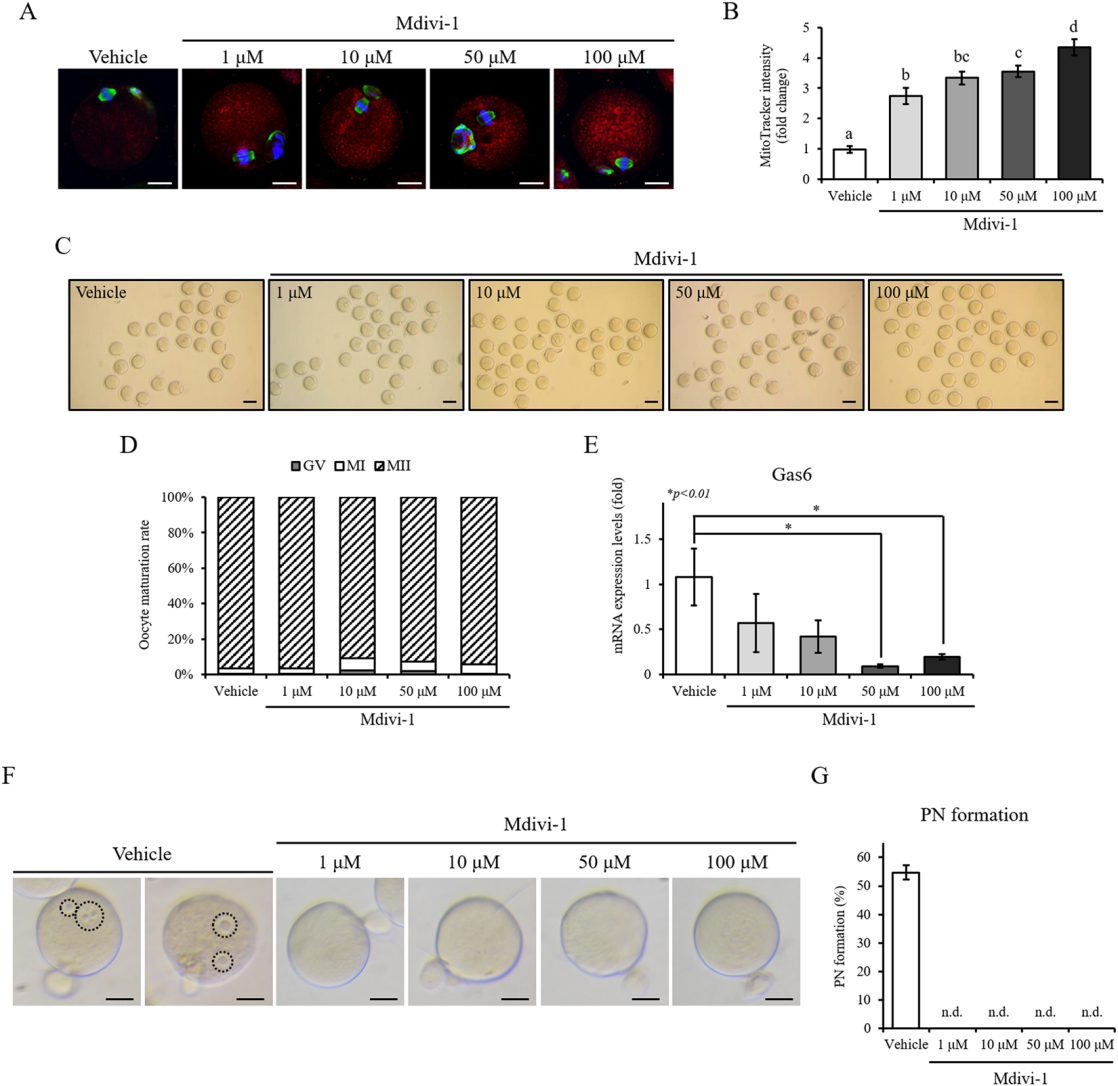
Reciprocal regulation of *Gas6* and mitophagy in oocyte. (**A**) The treatment with Mdivi-1 increased the mitochondrial content in oocytes. After *in vitro* oocyte maturation with Mdivi-1, oocytes were stained with MitoTracker (mitochondria, red) as well as with an antibody against α-TUBULIN (green). Oocyte DNA was also stained with DAPI (blue), and the oocytes were imaged by an LSCM. The scale bars indicate 20 μm. (**B**) MitoTracker intensity was calculated and graphical presentation of the increased mitochondrial content is shown in (**B**). Data are presented as the mean ± SEM. Different letters (a~b) indicate significant differences at *p* < 0.05. (**C**) Microphotographs of oocytes treated with vehicle (DMSO) as a control or with Mdivi-1 during oocyte maturation showed most of all oocytes developed to MII. The scale bars indicate 100 μm. (**D**) The *in vitro* maturation rate of mouse oocytes after Mdivi-1 stimulation was not changed. (**E**) Stimulation with Mdivi-1 resulted in the suppression of *Gas6* mRNA expression compared with the expression in the vehicle group (*p < 0.01). (**F**) Inhibition of mitophagy by Mdivi-1 led the impaired PN formation suggesting the similar phenomena observed in *Gas6* RNAi oocytes. GV oocytes were cultured in M16 medium without (vehicle) or with Mdivi-1 and then fertilized after *in vitro* culture. Black dot circles indicate PN formation. Scale bars indicate 50 μm. (**G**) Percentage of oocytes with PN formation after *in vitro* fertilization. n.d., not detected.

To address the effect of Mdivi-1, a mitochondrial division/mitophagy inhibitor, on oocyte nuclear maturation, mitochondrial quantity and expression of *Gas6* in oocytes, we analyzed the mitochondrial content in oocytes after Mdivi-1 treatment. As shown in Fig. 6A, the number of mitochondria, stained red, was increased by Mdivi-1 stimulation in a dose-dependent manner in the cytoplasm of MII oocyte (Fig. 6A and B). Our results revealed that the mitochondria were aggregated more commonly in *Gas6*-silenced MII oocytes (Fig. 3A) but were not aggregated in MII oocytes with Mdivi-1 stimulation (Fig. 6A). To further elucidate the relationship between the mitophagy suppression and oocyte maturation, the oocyte maturation rate was observed after treatment with Mdivi-1. Despite the increase in Mdivi-1 treatment in a dose-independent manner, oocyte maturation rates were not changed (Fig. 6C and D). These results suggest that the inhibition of mitophagy is not involved in oocyte nuclear maturation. We also found that the expression levels of *Gas6* in oocytes, which is implicated in the regulation of cytoplasmic maturation and mitochondrial function and/or content, was changed and decreased in a dose-dependent manner with Mdivi-1 treatment (Fig. 6E), suggesting that *Gas6* and mitophagy are interconnected and reciprocally regulate each other in oocytes.

To verify the effect of mitophagy inhibition by Mdivi-1 on fertilization, we added Mdivi-1 in the medium during oocyte maturation and then performed *in vitro* fertilization. Following *in vitro* fertilization, control oocytes (vehicle, open bar) exhibited a substantial percentage of PN formation after sperm penetration (Fig. 6F). However, similar to the effects of directly targeting *Gas6*, all MII oocytes with Mdivi-1 at any dose (1~100 μM) failed to *in vitro* fertilization with no PN formation (Fig. 6F). Taken together, mitochondrial accumulation due to suppression of mitophagy resulted in the insufficient cytoplasmic maturation and consequently the failure of fertilization.

## Discussion

**Figure 7:**
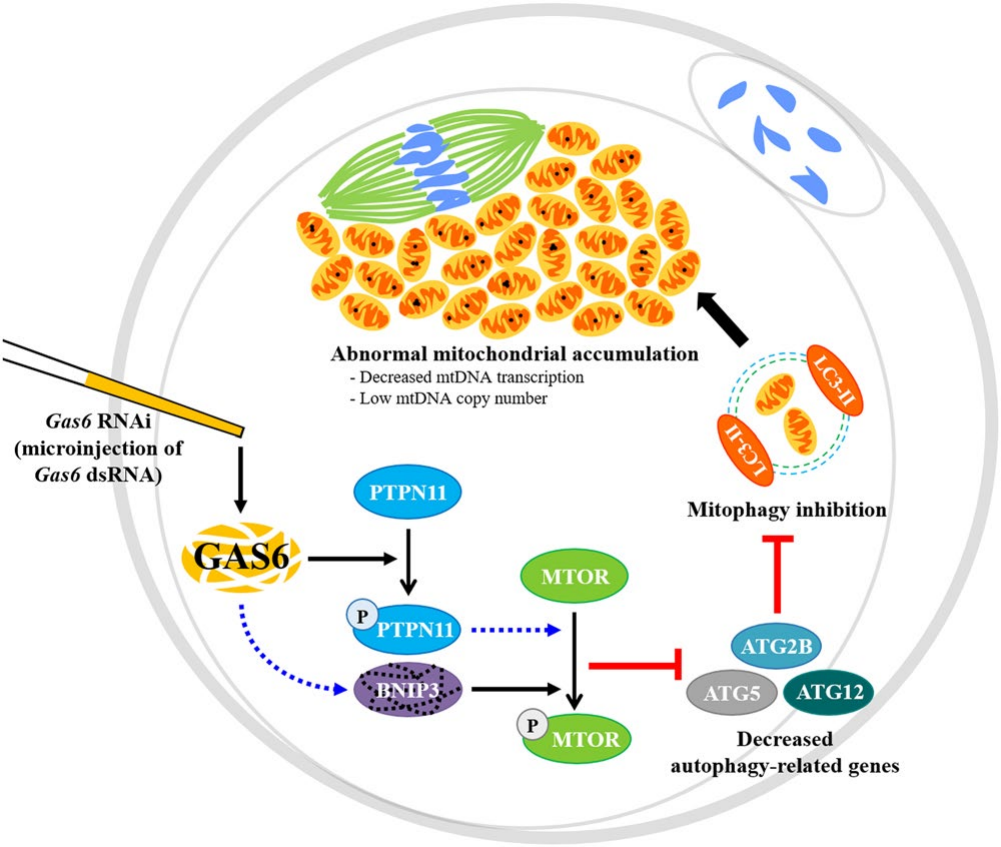
*Gas6* RNAi treatment induced mitochondrial dysfunction and accumulation by MTOR-dependent mitophagy suppression. *Gas6* silencing-induced PTPN11 activation and BNIP3 inhibition led the increased MTOR activity in oocytes. These oocytes exhibited a reduction in autophagy-related gene expression and autophagosome formation. In addition, *Gas6*-depleted oocytes had an abnormally large number of mitochondria as a result of the suppression of mitophagy. These mitochondria tended to accumulate around the spindle in the cytoplasm. Thus, the disruption of *Gas6* caused low mitochondrial quality and altered quantity through MTOR-dependent mitophagy inhibition and resulted in incompetent MII oocytes despite their normal appearance with polar body formation after maturation. The blue broken arrow indicates possible stimulatory pathways in present study and remains to be revealed with substantial experimental data.

In this study, we report a novel role for *Gas6* associated with mitochondrial quality and quantity through MTOR-dependent mitophagy signaling during oocyte maturation, and the regulation of mitophagy and *Gas6* expression are mutually interconnected. In *Gas6*-depleted MII oocytes, we observed an increased content of mitochondria and an aggregated and accumulated distribution of mitochondria from the meiotic spindle to the cortex. Disruption of *Gas6* resulted in stimulated PTPN11 downstream of *Gas6* and increased MTOR activity via reduction of BNIP3, autophagy-related genes and autophagosome formation-related genes, resulting in mitophagy suppression (Fig. 7). Interestingly, *Gas6* depletion led a reduction in mitochondrial transcription and mtDNA copy number, suggesting that GAS6 functions to maintain both the quality and quantity of mitochondria in oocytes for sufficient cytoplasmic maturation.

Autophagy, a very intriguing pathway, is regulated by multiple proteins, such as PI3K III, MTOR, MAPK and BNIP3^32^. During the past two decades, more than 30 autophagy-related genes involved in the regulation of the formation of autophagosome have been identified^22^. These proteins are also important for mitophagy. In the present study, we showed that mouse oocytes contain MTOR and BNIP3 proteins, the main regulators of mitophagy, and confirmed that those autophagy-related genes and LC3 are involved in autophagosome formation. Although each protein is essential for controlling mitophagy, the most commonly used marker for monitoring the mitophagic flux was the ratio of LC3-II/LC3-I^33^. Disruption of *Gas6* induced a significant decrease in the ratio of LC3-II/LC3-I. Ultimately, increased, accumulated, and aggregated mitochondria were found in the oocyte cytoplasm after *Gas6* RNAi. These results strongly indicate that GAS6 regulates mitochondrial distribution and quantity in oocytes via mitophagy.

Mitophagy, a selective form of autophagy, is responsible for eliminating dysfunctional or damaged mitochondria and maintaining the balance in mitochondria quality and quantity. MTOR is a key regulator of autophagy as well as mitophagy^30^. There is evidence that mitophagy requires the suppression of MTOR activity^25,30^. In the present study, we found that *Gas6*-depleted MII oocytes exhibit increased MTOR activity and, therefore, mitophagy inhibition. Upon rapamycin stimulation, the inhibition of MTOR activity in *Gas6*-silenced MII oocytes suppressed the downregulation of the autophagy-related genes and rescued the mitophagy caused by *Gas6* RNAi, which did not induce mitochondrial aggregation in the cytoplasm. Therefore, we concluded that GAS6 regulates mitophagy through a MTOR-dependent pathway, thereby efficiently safeguarding mitochondria in oocytes.

The regulation of mitochondrial dynamics is important for oocyte maturation. Mitochondria redistribute during oocyte maturation into regions requiring high ATP concentrations due to high metabolic demands^34^. During the first meiotic division, mitochondria aggregate around the spindle, migrate to the oocyte cortex in association with the spindle and then distribute asymmetrically, causing almost all mitochondria to remain in the oocyte and very few mitochondria to remain in the polar body^35^. During the second meiotic division, mitochondria aggregate around the spindle and then distribute throughout the cytoplasm^35,36^. We showed in this study that the mitochondria in *Gas6*-depleted MII oocytes also markedly accumulate and aggregate around the meiotic spindle; however, the mitochondria aggregates remained when the spindle formation was completed. In addition, previously, we found that the disruption of *Gas6* increases ATP production and the mitochondrial membrane potential^8^. However, *Gas6* RNAi does not affect oocyte nuclear maturation^7^. Despite the abnormal mitochondrial distribution, it is possible that the formation and separation of the meiotic spindle during nuclear maturation progressed normally in oocytes after *Gas6* RNAi because ATP was abundant in *Gas6*-depleted MII oocytes.

Oocyte maturation involves nuclear and cytoplasmic maturation, during which oocytes acquire fertilizability. Mitochondria play an important role in both processes since they provide the main supply of ATP^10^. It has been reported that mtDNA in oocytes is pivotal for healthy reproduction and that a lower mtDNA copy number can cause fertility problems^37^. Mouse oocytes containing as few as 50,000 copies of mtDNA can give rise to healthy embryos^38^. Prior to fertilization, the mtDNA copy number in oocytes needs to be amplified to sufficient levels. Mature oocytes contain at least 100,000 copies of mtDNA^38,39^, and we obtained the same results in this study. The mtDNA copy number in MII oocytes could be used as a marker of embryo developmental competence^11,13^. Moreover, studies have shown that a high mitochondrial content can increase the quality and competence of mature mammalian oocytes^11,38^. We did not determine how *Gas6* RNAi induced a lower mtDNA copy number in *Gas6*-silenced MII oocytes in this study, but it was approximately 50% that of the control oocytes, and we assume that the defective cytoplasmic maturation caused by *Gas6* silencing was due to this lower mtDNA copy number.

Mitochondrial dysfunction causes a decrease in oocyte quality and interferes with embryonic development, resulting in declining fertility. Recently, it has been reported that mitochondrial replacement therapy, a new gene-therapy technique, could lead to better fertility outcomes and a remarkable clinical amelioration of mitochondrial diseases^40^. The mitochondrial replacement therapy can be performed by three different processes, such as maternal spindle transfer^16,41^, polar body transfer^42^ and pronuclear transfer^43^. This technique in oocytes may safely prevent next-generation transmission of harmful mtDNA mutations^44^. The other method that improves mitochondrial function may be performed by using small molecules, such as resveratrol^45^, L-carnitine^46^ and coenzyme Q10^47,48^. In the present study, we also found that GAS6 is another avenue for safeguarding the mitochondrial quality and quantity in oocytes. Therefore, we propose that the discovery of *Gas6*-level regulation in oocytes may open a new method to improve oocyte mitochondrial function and its application in human IVF for oocyte competency. Although GAS6 seem to be appealing for improving fertility, well-designed clinical studies are necessary to test the therapeutic efficacy, safety and potential benefits, as well as its side effects for clinical application in IVF lab.

In conclusion, our findings clearly demonstrated that *Gas6* regulates mitophagy and safeguards mitochondrial activity through regulating central mitophagy-related genes, such as MTOR, BNIP3, ATG family and LC3, thereby resulting in fully competent oocytes (Fig. 7). We previously reported that GAS6 regulates mitochondrial function for ATP production and mitochondrial membrane potential^7^. It is noteworthy that mitochondrial accumulation with impaired mitophagy may induce the overactivation of mitochondria and lead to insufficient cytoplasmic maturation, with a negative impact on the remodeling sperm nucleus and pronuclear formation after fertilization. Taking these results together, we suggest that control of the GAS6 signaling network in oocytes may improve the decreased fertilizability caused by the deterioration of mitochondrial functions and/or contents.

## Methods

### Animals

Imprinting control region (ICR) mice, exclusively provided by Koatech (Pyeoungtack, Korea), were mated to produce embryos in the breeding facility at the CHA Research Institute of CHA University. All procedures described herein were reviewed and approved by the Institutional Animal Care and Use Committee of CHA University and were performed in accordance with the Guiding Principles for the Care and Use of Laboratory Animals.

### Reagents

Chemicals and reagents were obtained from Sigma-Aldrich (St. Louis, MO, USA) unless otherwise noted.

### Oocyte isolation

To isolate germinal vesicle (GV) oocytes, three-week-old female ICR mice were injected with 5 IU pregnant mare serum gonadotropin and sacrificed 46 hours later. Isolated ovaries were punctured in M2 medium containing 0.2 mM 3-isobutyl-1-methyl-xanthine (IBMX), and cumulus-oocyte complexes (COCs) were collected. Cumulus cells were mechanically retrieved from oocytes by repeated pipetting via a fine-bore pipette. To obtain oocytes in other stages, GV oocytes were cultured in M16 medium without IBMX in 5% CO_2_ at 37 °C. Isolated oocytes were snap-frozen and stored at −80 °C prior to RNA isolation.

### Microinjection and *in vitro* culture

For RNAi experiments, we prepared *GFP* and *Gas6* dsRNA as previously described^7^. GV oocytes were microinjected with dsRNA in M2 medium containing 0.2 mM IBMX; 10 pl of dsRNA was microinjected into the oocyte cytoplasm using a constant flow system (Transjector; Eppendorf, Hamburg, Germany). The oocytes were cultured in M16 medium containing 0.2 mM IBMX for 8 hours, followed by culture in M16 alone for 16 hours in 5% CO_2_ at 37 °C. After the RNAi experiments were completed, *in vitro* maturation rates and morphological changes were recorded as previously described^7^.

### Library preparation and RNA-Seq (QuantSeq)

Total RNA (300 MII oocytes) was isolated using a RNeasy mini kit (Qiagen, Inc., Valencia, CA, USA). RNA quality was assessed by an Agilent 2100 bioanalyzer using an RNA 6000 Nano Chip (Agilent Technologies, Amstelveen, Netherlands), and RNA quantification was performed using an ND-2000 Spectrophotometer (Thermo, Inc., Waltham, MA, USA). For control and test RNAs, the library construction was performed using a SENSE 3′ mRNA-Seq Library Prep Kit (Lexogen, Inc., Vienna, Austria) according to the manufacturer’s instructions. In brief, each 500 ng sample of total RNA was prepared, an oligo-dT primer containing an Illumina-compatible sequence at its 5′ end was hybridized to the RNA, and reverse transcription was performed. After degradation of the RNA template, second-strand synthesis was initiated by a random primer containing an Illumina-compatible linker sequence at its 5′ end. The double-stranded library was purified by using magnetic beads to remove all reaction components. The library was amplified to add the complete adapter sequences required for cluster generation. The finished library was purified from PCR components. High-throughput sequencing was performed as single-end 75 sequencing using NextSeq. 500 (Illumina, Inc., San Diego, CA, USA).

### Data analysis

SENSE 3′ mRNA-Seq reads were aligned using Bowtie2 version 2.1.0^49^. Bowtie2 indices were generated from either the genome assembly sequence or the representative transcript sequences for aligning to the genome and transcriptome. The alignment file was used for assembling transcripts, estimating their abundances and detecting the differential expression of genes. Differentially expressed genes were determined based on counts from unique and multiple alignments using EdgeR within R version 3.2.2 (R development Core Team, 2011) using Bioconductor version 3.0^50^. The read count data were processed based on the global normalization method using the Genowiz™ version 4.0.5.6 (Ocimum Biosolutions, Hyderabad, India). We analyzed differentially expressed genes and generated a scatter plot to compare expression values (Fig. S1). Gene classification was based on searches performed in the DAVID (http://david.abcc.ncifcrf.gov/) and MEDLINE databases (http://www.ncbi.nlm.nih.gov/).

### Rapamycin and Mdivi-1 treatment of oocytes

An experiment of rescued mitophagy in *Gas6*-depleted MII oocytes was performed in the presence or absence of rapamycin, a specific inhibitor of MTOR. After RNAi, oocytes were cultured in M16 medium containing 1 nM rapamycin, followed by staining of mitochondria (MitoTracker) and Western blot analysis of MTOR and p-MTOR.

For analysis of oocyte nuclear maturation by mitochondrial division/mitophagy inhibitor Mdivi-1, *in vitro* maturation rates and morphological changes were recorded after incubation with 1 μM, 10 μM, 50 μM and 100 μM Mdivi-1 for 16 hours. After treatment, the oocytes were washed thoroughly and prepared for qPCR. Oocytes in the vehicle group were treated with the same concentration of DMSO in M16 medium.

### *In vitro* fertilization (IVF)

Sperm were collected from the caudal epididymides of 8-week-old male ICR mice (Koatech). The sperm were incubated in M16 medium for 1 hour to allow capacitation. After the zona pellucida (ZP) was removed using Tyrode’s solution (pH 2.5), ZP-free MII oocytes were then placed in a 200 μl droplet of M16 medium under mineral oil and inseminated with 2.5 × 10^4^/ml sperm. After 2 hours, the oocytes were washed to remove unbound sperm and cultured in M16 medium for 5 hours (37 °C, 5% CO_2_) to observe PN formation.

### Messenger RNA isolation

Oocyte mRNA was isolated using a Dynabeads mRNA DIRECT kit (Dynal Biotech ASA, Oslo, Norway) according to the manufacturer’s instructions. Briefly, oocytes were suspended with lysis/binding buffer and mixed with prewashed Dynabeads oligo dT_25_. After RNA binding, the beads were washed with buffer A twice, followed by buffer B, and mRNA was eluted with Tris-HCl by incubation at 72 °C. Purified mRNA was used as a template for reverse transcription with an oligo (dT) primer according to the MMLV protocol.

### Quantitative real-time RT-PCR (qPCR)

qPCR was carried out with a single-oocyte-equivalent amount of cDNAs and gene specific primers (Table 1), as previously described^7^, using an iCycler iQ™ Detection System (Bio-Rad). The iQ SYBR Green Supermix PCR reagents (Bio-Rad) were used to monitor amplification, and the results were analyzed using iCycler iQ™ proprietary software. The melting curves were used to identify any nonspecific amplification products. *H1foo* was used as a control. The relative expression levels of the target genes were evaluated using the comparative C_T_ method, and all experiments were repeated at least three times.

**Table 1:** Primer sequences and RT-PCR conditions.

Gene symbol	Description	Accession numbers	Primer sequence^a^	Annealing temperature	Product size
*Gas6*-A^b^	Growth arrest specific 6	NM_019521.2	For-CCGTGATTAGACTACGCTTC Rev-AGTTGAGCCTGTAGGTAGCA	60 °C	561 bp
*Gas6*-B^c^	Growth arrest specific 6	NM_019521.2	For-AAAGGGCCAGAGTGAAGTGA Rev-TTTTCCCGTTTACCTCCAGA	60 °C	175 bp
*Atg2b*	Autophagy related 2B	NM_029654.4	For-CGTAAAGCCCATTCCAACAT Rev-AGATTTGGCTCCTTTGAGGT	60 °C	275 bp
*Atg5*	Autophagy related 5	NM_053069.5	For-ATGTGCTTCGAGATGTGTGG Rev-CAGGGGTGTGCCTTCATATT	60 °C	215 bp
*Atg7*	Autophagy related 7	NM_001253717.1	For-ATGCCAGGACACCCTGTGAACTTC Rev-ACATCATTGCAGAAGTAGCAGCCA	60 °C	351 bp
*Atg12*	Autophagy related 12	NM_026217.3	For-TTCGGTTGCAGTTTCGCC Rev-CCATGCCTGTGATTTGCAGTA	63 °C	311 bp
*Becn1*	Beclin1	NM_019584.3	For-TTTGACCATGCAATGGTAGC Rev-TGGTCAGCATGAACTTGAGC	60 °C	211 bp
*Bnip3*	BCL2/adenovirus E1B interacting protein 3	NM_009760.4	For-AGCTTTGGCGAGAAAAACAG Rev-TCCAATGTAGATCCCCAAGC	60 °C	297 bp
*Esr2*	Estrogen receptor 2 (beta)	NM_207707.1	For-TGTGTGTGAAGGCCATGATT Rev-TCATGCTGAGCAGATGTTCC	60 °C	246 bp
*Map1lc3a*	Microtubule-associated protein 1 light chain 3 alpha	NM_025735.3	For-AGCTTCGCCGACCGCCGTAAG Rev-CTTCTCCTGTTCATAGATGTCAGC	63 °C	276 bp
*Map1lc3b*	Microtubule-associated protein 1 light chain 3 beta	NM_026160.4	For-CGGAGCTTTGAACAAAGAGTG Rev-TCTCTCACTCTCGTACACTTC	63 °C	279 bp
*Mtor*	Mechanistic target of rapamycin (serine/threonine kinase)	NM_020009.2	For-GGAGGCTGATGGACACAAAT Rev-CTCCACTTGGGTTGGAACAT	60 °C	293 bp
*Phyhipl*	Phytanoyl-CoA hydroxylase interacting protein-like	NM_178621.4	For-GTTTTTGAGCCCAAGGACTG Rev-TTTGTGCTGGTTCCGATAGA	60 °C	211 bp
*Ptpn11*	Protein tyrosine phosphatase, non-receptor type 11	NM_011202.3	For-GGACAGGAACCTTCATTGTG Rev-TTGCTTTTCTGCTCCTCCTC	60 °C	214 bp
*Tomm7*	Translocase of outer mitochondrial membrane 7 homolog	NM_025394.3	For-GCAAAGAAGCCAAACAGAGG Rev-TCTGCACCCCTTGTAAATCC	60 °C	109 bp
*H1foo*	H1 histone family, member O, oocyte-specific	NM_138311	For-GCGAAACCGAAAGAGGTCAGAA Rev-TGGAGGAGGTCTTGGGAAGTAA	60 °C	378 bp
*GFP* ^b^	Green fluorescent protein	KF111246.1	For-TGTCCCAATTCTTGTTGAAT Rev-TTGTCTGGTAAAAGGACAGG	60 °C	561 bp

^a^For, Forward; Rev, Reverse.

^b^Primers were used for preparation of *Gas6* dsRNA and *GFP* dsRNA, respectively.

^c^Different set of primers used for confirming the knockdown of *Gas6* transcripts after RNAi treatment.

### Measurement of the mitochondrial DNA copy number

Mitochondrial DNA extraction in 50 MII oocytes after *GFP* RNAi or *Gas6* RNAi was performed using a mitochondrial DNA isolation kit (Abcam, Cambridge, MA, USA). For mtDNA content analysis in oocytes, we selected a region of the mouse mitochondrial *Nd1* gene corresponding to bases 2929–3128 (Accession number NC_005089.1). The mtDNA copy number was determined by performing qPCR using an iCycler iQ™ Detection System with the primer set (5′-CAATACGCCCTTTAACAACC-3′ and 5′-TTTGGAGTTTGAGGCTCATC-3′) and iQ SYBR Green Supermix PCR reagents. The mtDNA content in a single oocyte was extrapolated from the standard curve and calculated according to the target PCR product length. A standard curve was generated for each run using 10-fold serial dilutions representing the copy number of the external standard. The external standard was the PCR product of the corresponding gene cloned into a vector using the Zero Blunt TOPO PCR cloning kit (Invitrogen, Carlsbad, CA, USA), and the PCR product was sequenced for confirmation before use. All samples were analyzed in triplicate.

### Western blotting

To confirm protein expression, Western blotting was performed as previously described^7^. Briefly, a protein extract was separated using 6%, 10% or 15% SDS-PAGE and transferred onto a PVDF membrane (Amersham Biosciences, Piscataway, NJ). After blocking, the membrane was incubated with antibodies against GAS6 (1:1000), BNIP3 (1:1000; #3769, Cell Signaling Technology, Danvers, MA, USA), LC3 (1:1000; ab48394, Abcam), MTOR (1:1000; #2972, Cell Signaling Technology), p-MTOR (1:1000; #2971, Cell Signaling Technology), PTPN11 (1:1000; #3752, Cell Signaling Technology), p-PTPN11 (1:1000, #3751, Cell Signaling Technology), and α-TUBULIN (1:2000; sc-8035, Santa Cruz Biotechnology), followed by incubation with HRP-conjugated anti-goat IgG (1:2000; A5420), anti-rabbit IgG (1:5000; #7074, Cell Signaling Technology, Danvers, MA, USA) or anti-mouse IgG (1:2000; A2554). Bound antibodies were detected using an enhanced chemiluminescence detection system (Amersham Biosciences) according to the manufacturer’s instructions.

### Staining of mitochondria

To evaluate the distribution of mitochondria, oocytes were stained using MitoTracker Orange CMTMRos (Molecular Probes). MitoTracker, which fluoresces orange, was used at a concentration of 300 nM in M16 supplemented with 0.3% BSA for 30 minutes at 37 °C in the dark. After washing, the oocytes were fixed and immunofluorescently stained with an anti-α-TUBULIN antibody and then counterstained with DAPI. The stained oocytes were stored below 4 °C until confocal microscopy was performed. The oocytes were examined using a laser scanning confocal microscope (LSCM; Leica, Wetzlar, Germany). A minimum of 30 oocytes per RNAi group was examined by an LSCM. For the fixed MitoTracker measurements, the signal intensities for each oocyte were calculated using Leica Application Suite Advanced Fluorescence (LAS AF) software. Data represent the average fold change from three experiments.

### Immunofluorescence staining

Denuded oocytes were placed in PBS containing 0.1% polyvinyl alcohol (PBS-PVA), 4% paraformaldehyde, and 0.2% Triton X-100 and then fixed for 40 minutes at room temperature (RT). After the oocytes were washed in PBS-PVA, fixed oocytes were blocked with 3% BSA-PBS-PVA for 1 hour and then incubated with mouse anti-α-TUBULIN antibody (1:100) at 4 °C overnight. After the oocytes were washed, they were incubated with an Alexa Fluor 488-conjugated secondary antibody (Molecular Probes, Eugene, OR, USA) for 1 hour at RT.

### Statistical analysis

Data were derived from at least three separate and independent experiments and expressed as the mean ± SEM. The *p* values were calculated based on a paired *t*-test of the means from the *GFP* RNAi group and the *Gas6* RNAi group, and *p* < 0.05 was considered statistically significant.

## Supplementary information

Supplementary information file 1

Supplementary information file 2
