# Supplementary material for: Gas6 is a reciprocal regulator of mitophagy during mammalian oocyte maturation
Source: Sci Rep. 2019 Jul 17;9:10343. doi: 10.1038/s41598-019-46459-3 (PMC6637152; doi:10.1038/s41598-019-46459-3)
Supplement: Supplementary file 1 — Supplementary information file 1 [file 41598_2019_46459_MOESM1_ESM.pdf]

Gas6 is a reciprocal regulator of mitophagy during mammalian oocyte maturation

Kyeoung-Hwa Kim, Eun-Young Kim, Jung-Jae Ko, Kyung-Ah Lee*

Institute of Reproductive Medicine, Department of Biomedical Science,

College of Life Science, CHA University,

Pangyo-Ro 335, Bundang-gu, Seongnam-si, Gyeonggi-do, 13488, Korea

* Correspondence should be addressed to: Kyung-Ah Lee, leeka@cha.ac.kr

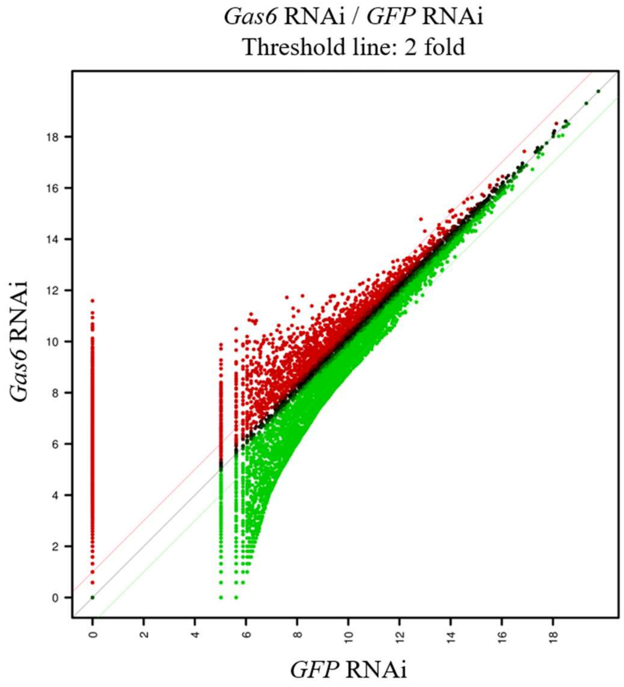

Fig. S1. Scatter plot of differentially expressed genes in oocytes treated with *GFP* RNAi vs. *Gas6* RNAi. Red points indicate genes upregulated in *Gas6* RNAi oocytes relative to *GFP* RNAi oocytes, green points represent genes downregulated in *Gas6* RNAi oocytes relative to *GFP* RNAi oocytes, and black points represent genes that either showed no differences or exhibited changes below 2-fold.

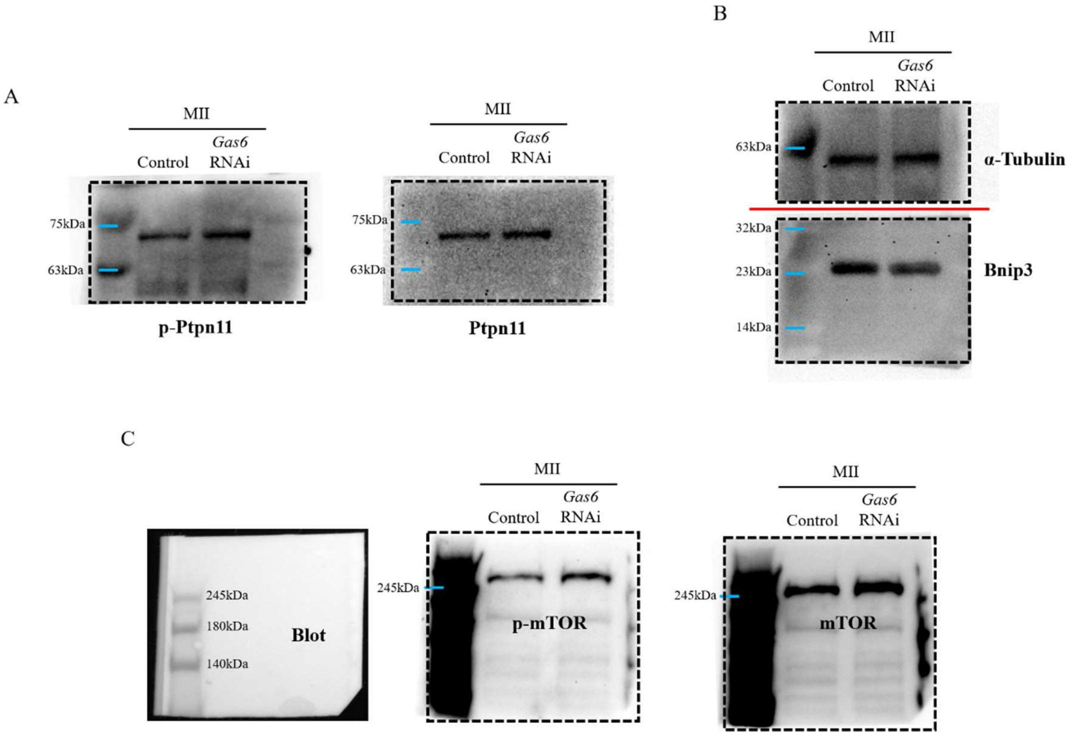

Fig. S2. Full-length blots corresponding to Figure 2C. (**A**) Protein expression of p-PTPN11 and PTPN11 in *Gas6*-silenced MII oocytes. Blot probed for p-PTPN11 and then for PTPN11 antibody. The size (kDa) of the protein markers are indicated on the left side of the figures. Black hatched lines delineate the approximate boundaries of the original membrane. (**B**) Expression of BNIP3 protein in *Gas6*-depleted MII oocytes. The α-TUBULIN was used as a loading control. Red line shows where gels were cut before immunoblotting with indicated antibody. Black hatched lines delineate the approximate boundaries of the original membrane. (C) The figures are showing full length original blot images (left). Western blot analysis of p-MTOR after *Gas6* RNAi (center). Blots were stripped and re-probed with MTOR (right). Black hatched lines delineate the approximate boundaries of the original membrane.

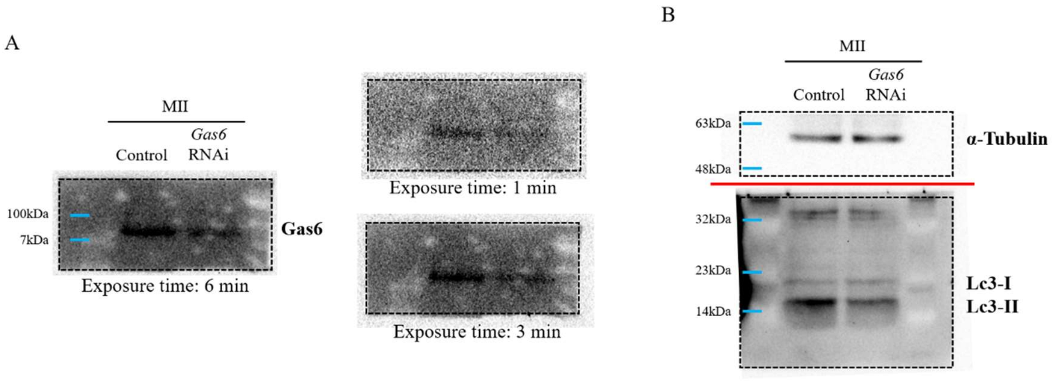

Fig. S3. Full-length blots corresponding to Figure 3C. (**A**) *Gas6* disrupts GAS6 protein expression, as confirmed by Western blot analysis. Signals were detected using the ChemiDoc XRS+ imaging system, exposed at the indicated times. The size (kDa) of the protein markers are indicated on the left side of the figures. Black hatched lines delineate the approximate boundaries of the original membrane. (**B**) Expression of LC3 proteins in *Gas6*-depleted MII oocytes. The α-TUBULIN was used as a loading control. Red line shows where gels were cut before immunoblotting with indicated antibody. Black hatched lines delineate the approximate boundaries of the original membrane.

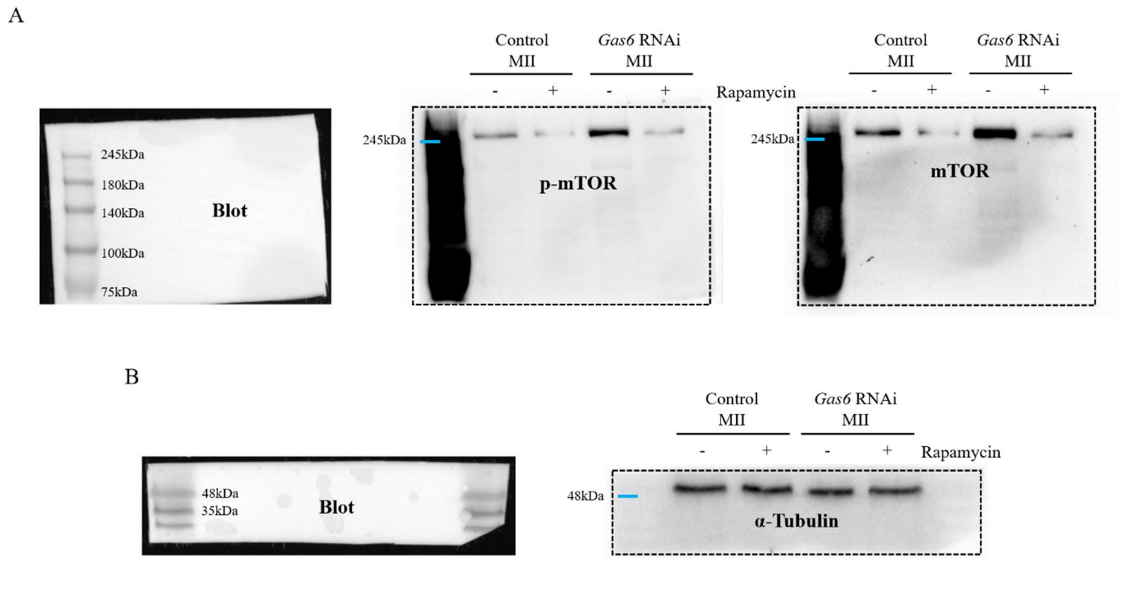

Fig. S4. Full-length blots corresponding to Figure 5A. (**A**) The figures are showing full length original blot images (left). Upon rapamycin stimulation, protein expression of p-PTPN11 (center) and PTPN11 (right) in *Gas6*-silenced MII oocytes. Blot probed for p-PTPN11 and then for PTPN11 antibody. The size (kDa) of the protein markers are indicated on the left side of the figures. Black hatched lines delineate the approximate boundaries of the original membrane. (**B**) The figures are showing full length original blot images (left). The α-TUBULIN was used as a loading control.

Table S1. Top 20 differential expressed genes.

Gene symbol	log2 ratio (*Gas6* RNAi/*GFP* RNAi)	Up/down regulation	Description
Uqcc1	29.4489795918367	Up	Ubiquinol-cytochrome C reductase complex chaperone
Aqp9	29.2448979591837	Up	Aquaporin 9
Tpm1	26.1928571428572	Up	Tropomyosin 1, alpha
Runx2	23.3856209150327	Up	Runt related transcription factor 2
Musk	21.3609467455621	Up	Muscle, skeletal, receptor tyrosine kinase
Cdk13	21.2448979591837	Up	Cyclin-dependent kinase 13
Eda	21.0365853658536	Up	Ectodysplasin-A
Ash2l	20.3170731707317	Up	Ash2 (absent, small, or homeotic)-like (Drosophila)
Spata7	17.7575757575758	Up	Spermatogenesis associated 7
Dnmt3b	17.4651162790698	Up	DNA methyltransferase 3B
Lsp1	11.8870151770658	Up	Lymphocyte specific 1
Wdr47	11.3827751196172	Up	WD repeat domain 47
Ewsr1	10.9738562091503	Up	Ewing sarcoma breakpoint region 1
Dctn3	10.3543689320388	Up	Dynactin 3
Tle4	10.1639871382637	Up	Transducin-like enhancer of split 4, homolog of Drosophila E (spl)
Pus7	9.83730158730159	Up	Pseudouridylate synthase 7 homolog (S. cerevisiae)
Rmi1	9.57522123893806	Up	RMI1, RecQ mediated genome instability 1, homolog (S. cerevisiae)
Sema6d	9.34343434343434	Up	Sema domain, transmembrane domain (TM), and cytoplasmic domain, (semaphorin) 6D
H2-T10	8.84310018903592	Up	Histocompatibility 2, T region locus 10
Setdb1	8.69032258064516	Up	SET domain, bifurcated 1
